# Body composition and energy expenditure in anorexia nervosa: preliminary data of outpatients with recovering and active disease

**DOI:** 10.1186/s40337-022-00702-x

**Published:** 2022-11-16

**Authors:** Tâmara Oliveira dos Reis, Fernanda de Magalhães Oliveira, Fabiana Martins Kattah, Natalia Fenner Pena, Maria Marta Sarquis Soares, Henrique Oswaldo da Gama Torres

**Affiliations:** 1 Adult Health Post-Graduate Program, Faculdade de Medicina, Universidade Federal de Minas Gerais, Belo Horizonte, Brazil; 2 Internal Medicine Departament, Faculdade de Medicina, Universidade Federal de Minas Gerais, Belo Horizonte, Brazil; 3 Núcleo de Investigação de Anorexia E Bulimia (NIAB), Hospital das Clinicas, Universidade Federal de Minas Gerais, Belo Horizonte, Brazil; 4 Nutrition Departament, Escola de Enfermagem, Universidade Federal de Minas Gerais, Belo Horizonte, Brazil

**Keywords:** Anorexia Nervosa, Body composition, Bioelectrical impedance, BMI, Resting metabolism, Phase angle

## Abstract

**Background:**

In Anorexia Nervosa (AN) recovery, body mass index (BMI) may not reflect body composition. To investigate recovery, bioelectrical impedance (BIA) parameters and energy expenditure were investigated in patients with active and recovering AN, with emphasis on phase angle (PA), a BIA parameter.

**Methods:**

BMI, PA, indirect BIA parameters (fat free mass, fat mass, total body water, fat free mass index, fat mass index) and resting metabolic rate (RMR) were obtained. Data from subjects distributed to active AN (ANact, *n* = 9), recovered AN (ANrec, *n* = 9) and healthy individuals (HI) (*n* = 16) were compared employing univariate methods and ordinal logistic regression.

**Results:**

In univariate comparison, the BMI would not distinguish recovered individuals; this distinction was observed for the PA (*p* =  < 0,001). PA showed a good capacity to discriminate, between ANrec and HI (AUC = 0.792; CI = 0.564- 1.000; *p* = 0.017). In 2 models of ordinal logistic regression PA (OR = 0.123; 95% CI 0.030; 0.503 and OR = 0.091; 95% CI 0.016; 0.528) remained as a significant independent variable, indicating that increases in PA are related to higher probabilities of moving from ANact, to ANrec and to HI group. Bivariate regression indicated the presence of a relationship between PA and (*R*2 = 0.266, *p* = 0.002).

**Conclusions:**

Changes in body composition and energy expenditure were observed in recovered anorexics with normal BMI. PA can play an important role in the assessment of recovering anorexic patients.

## Plain English summary

In recovery from anorexia nervosa, the body mass index does not reflect changes in body composition. Energy expenditure can change according to body composition in an anorexia nervosa and normal individuals. To investigate distinctive parameters in recovering Anorexia Nervosa patients, we evaluated body mass index and other indices obtained from body measurements by bioimpedance among patients with anorexia nervosa with low weight and in the weight recovery phase compared with a group of normal weight women without anorexia nervosa. In conclusion, we observed that one of these indexes, the phase angle can play an important role in the assessment of nutritional status in recovering anorexia nervosa.

## Background

Recovery criteria for Anorexia Nervosa (AN), based on body weight and Body Mass Index (BMI) normalization and improvement of symptoms related to body image and eating [1–3] are broad and not very sensitive, and vary among studies. Several combinations of these criteria are observed during recovery and consensus regarding their definition seems difficult to be achieved [4].

Guidelines consider weight regain and body composition restoration as main objectives of nutritional treatment of AN [5]. Currently, the Body Mass Index (BMI) represents the parameter for evaluating the nutritional status most widely recommended [1].

Among psychiatric diseases, AN shows the highest mortality rate, with relapses between 12 and 27% [6, 7]. About 40% of patients achieve recovery, 30% show improvement, 20% remain chronically affected and 10% die [8]. These data show the importance of understanding and monitoring recovery

Bioelectrical Impedance (BIA) is widely used to determine body composition in AN [9–11]. Single frequency BIA (50 kHz) provides various parameters, including FFM, FM, total body water (TBW) and phase angle (PA) [12]. PA has been investigated in AN as a prognostic indicator and in the evaluation of cell integrity among different causes of low weight, such as constitutional thinness and AN [13].

Changes in body composition in AN are characterized by drastic changes in fat mass (FM), fat-free mass (FFM) and bone mass [11, 14, 15]. Analyses of these compartments can add information about disease stage, considering that they may not be affected to the same extent, depending on the presence of purgatory practices such as physical activity, vomiting, use of laxatives, on the characteristics of the metabolism in starvation and, possibly, on the stage of recovery [6].

Despite its role in diagnostic definition, BMI does not indicate which body compartment is affected [15]. Although it is recognized that FFM and FM are deeply affected during the active phase of AN, they cannot be used for the purpose of comparing individuals, since they vary according to physical constitution. For this reason, other easily obtainable measures, including BIA direct measures and indirect parameters, should be sought to assess the stage of anorexia nervosa.

PA has been studied as a predictor of prognosis when assessing cellular integrity between different forms of low weight, such as constitutional thinness and AN [15]. In addition to the observed relationship between PA and body composition in individuals with AN, PA has also been related to energy expenditure [16].

In AN, resting metabolic rate (RMR) undergoes changes mainly due to the loss of muscle mass, which is one of its main determinants [16–18]. In addition, important reductions in RMR have been observed in AN [19].

Detection of changes in body composition by BIA direct and indirect indices could provide important support for clinical monitoring and definition of the stage of the disease. The assessment of BIA indirect indices should include those that take the patient’s constitution into account, such as the fat free mass index (FFMI) and fat mass index (FMI) in order to overcome BMI’s lack of sensitivity and its limitation in distinguishing which compartment is affected [20, 21]. RMR, due to its changes along AN recovery and to its relationship to FFM, may add to the evaluation of AN stages.

The preliminary data presented in this study are part of an effort to investigate whether BIA direct and indirect indices display distinctive characteristics in the group of recovered individuals. A special emphasis was placed on the PA, as it constitutes a direct and simple parameter, without the need for formulas obtained by regression models. Additionally, behavior of RMR in recovered patients and its relation with BIA parameters were evaluated as an additional attempt to characterize recovery.

## Methods

From May to November, 2019, patients followed at an outpatient eating disorders’ clinic operating as a statewide public point of reference in an university hospital were invited to take part in the study. Patients underwent a multidisciplinary treatment on weekly basis that included physicians (psychiatrists and nutrition specialist), psychologists and dietitians, scheduled according to clinical needs. Residents, students, and trainees in the areas of pediatrics, endocrinology, psychology, psychiatry and nutrition also attended the service.

Participants were informed about the objectives and the study protocols before signing the Informed Consent Form (ICF). Patients under 18 and their adult legal representative also signed the consent form previously approved by the University Ethics Committee, under the number CAAE 53641815.6.0000.5149.

AN diagnosis and recovery criteria followed the Diagnostic and Statistical Manual of Mental Disorders, 5th edition [1]. Patients with thyroid disease assessed by thyroid stimulating hormone (TSH) were excluded from the study due to changes in energy expenditure.

University students and employees composed the control group, according to the following inclusion criteria: absence of AN, female gender, BMI between 18.5 and 24.9 kg/m^2^, regular menstrual cycles, and absence of disease affecting energy metabolism.

Three study groups were defined: Active AN (ANact), recovered AN (ANrec) and healthy individuals (HI). Inclusion criteria in the ANact group were BMI below 18.5 kg/m^2^ and/or weight ad equacy below 85% of ideal weight, with the presence of

key symptoms as defined by DSM-5 (fear of gaining weight and disturbed body image). ANrec group inclusion criteria included previous AN according to the DSM-5 and BMI above 18.5 kg/m^2^, and/or weight adequacy above 85% of the ideal weight, maintained for a sustainable period, in addition to partial or total remission of the key symptoms as highlighted above. For this last parameter, the evaluation of the multidisciplinary team that included psychologists and psychiatrists was considered.

Nutritional assessment was carried out individually in a silent environment, at room temperature of 22 ºC to 25 ºC by the researcher and two trained assistants.

Body weight and BMI measurement were performed with a mechanical scale calibrated with ± 0.1 kg precision coupled to a stadiometer with ± 0.1 cm precision for height measurement.

Nutritional status was based on the BMI calculated according to the standard formula: weight (kg)/height (m^2^) [22]. Subjects were considered eutrophic when BMI values were between 18.50 kg/m^2^ and 24.99 kg/m^2^ for adults [22] or Z score ≥ − 2 and

<+1 for adolescents from 10 to 19 years old [23].

Body composition was assessed by a Quantum X® [24] bioimpedance device of low intensity (800 μA) and single frequency (50 kHz). Prior to the BIA exam, patients were instructed to undergo a food and beverage fast and to refrain from drinking alcohol and from performing physical activity. BIA was not performed during menstrual cycles. BIA provided the values for resistance (R) and reactance (Xc) by the Body Composition Program and calculation of PA was performed according to the formula: PA = arc-tangent (Xc/R) [16, 24].

BIA derived equations provided values for TBW and FFM. The formulas for FFM and TBW were: FFM (women) = − 4.104 + 0.518 x RI + 0.231 x Weight + 0.130 x Xc; TBW (women) = 0.434 x weight + 6.326 [16]. FM was calculated from FFM and TBW. FM and FFM indices were calculated from the equation: FMI = fat mass (kg)/height^2^ (m^2^); FFMI = fat free mass (kg)/height^2^ (m^2^) [24]. Normal values for FFMI are between 15.0 kg/ m^2^ - 16.6 kg/m^2^ [20].

For IC, the MetaCheck® calorimeter was employed. At a comfortable room temperature of 22 to 25ºC. All participants were instructed to fast for at least 5 hours before testing. The device estimates the individual's resting metabolic rate in kcal/day from the VO2, considering that each calorie consumed needs an amount of the supplied oxygen to be converted into energy, according to the equation by Weir [25]. Measurements were performed at the same time of the day to avoid fluctuations in weight, thus affecting body composition and RMR.

Data were analyzed with the software Statistical Package for Social Science (SPSS) version 25. Variables were classified into three categories: 1) anthropometric: BMI (kg/m^2^); 2) direct BIA parameters: R (Ω), Xc (Ω) and PA(^o^); 3) indirect BIA parameters FFM (kg), FFM (%), FM (kg), FM (%), FFMI (kg/m^2^), FMI(kg/m^2^), TBW(kg) and TBW(%).

Differences among groups (ANact, ANrec and HI) were analyzed by comparison of means for variables with normal distribution (ANOVA with *post hoc’*Bonferroni correction) or comparison of medians for variables with non-normal distribution (Kruskal Wallis, followed by Mann-Whitney with Bonferroni correction, in case of statistical significance). Significance threshold was set at 0.05. ROC (receiver operating characteristic) curves were constructed for the studied variables, with ANact x ANrec and ANrec x HI as comparison groups.

Considering that the three study groups presented progressive and ordered values in their anthropometric and BIA variables (Table 1), an attempt to establish correlations between the independent variables and classification in each study group was made by means of an ordinal regression. The ordered categories ANact (outcome 2), ANrec (outcome 1) or HI (outcome 0) were considered dependent variables. The variables listed in Table 1 were used as independent variables. As a first step, separate univariate regressions were performed for each variable and those with *p* < 0.2 were chosen to enter the models. As a second step, Spearman's correlation analysis was performed with the variables obtained in the first step in order to select groups of variables in which all correlation coefficients were below 0.7. Separate ordinal regression models were performed for each one of these groups. Model fit was tested by the Deviance chi-square test and assumption of proportional odds was tested by the test of parallel lines. BMI was not included among independent variables due to its determinant role in the definition of the 3 study groups. Ordinal regression results were presented as odds ratio.

**Table 1:** Comparison of BMI, duration of AN and age, weight, direct and indirect bioimpedance parameters among groups

Variables	ANact (*n* = 9)	ANrec (*n* = 9)	HI (*n* = 16)	*p*	ANact *x*	ANrec *x*	ANact *x*
					HI	HI	ANrec
BMI*	15.92 ± 2.58	20.32 ± 2.21	22.24 ± 1.75	< 0.001	< 0.001***	0.112***	< 0.001***
(Kg/m^2^)							
Duration**	48.0(20.5;156.0)	28.5(12.5; 57.0)		0.47			
(months)							
Age** (years)	23.0(19.0; 35.0)	22.0(17.0; 24.0)	23.5(22.0; 31.0)	0.335			
Weight* (kg)	39.4 ± 5.9	51.4 ± 5.3	60.3 ± 6.7	0			
R**(Ω)	749.0(626.0; 832.0)	614.0(582.5; 655.5)	528.5(508.5; 591.0)	0.002	0.003****	0.009****	0.047****
Xc**(Ω)	69.0(47.5; 74.5)	61.0(59.5; 73.5)	64.0(60.5; 69.8)	0.898	–	–	–
PA** º	4.7(4.1; 5.9)	5.8(5.5; 6.8)	6.9(6.6; 7.1)	< 0.001	< 0.001****	0.017****	0.033****
FFM* (kg)	31.01 ± 4.56	37.59 ± 1.89	44.56 ± 4.64	< 0.001	< 0.001***	< 0.001***	0.005***
FM* (kg)	8.38 ± 3.59	13.86 ± 4.75	15.78 ± 5.06	0.002	0.002***	0.982***	0.053***
FFM** (%)	0.82(0.72; 0.86)	0.73(0.68; 0.77)	0.73(0.70; 0.77)	0.18	–	–	–
FM** (%)	0.18(0.14; 0.28)	0.27(0.23; 0.32)	0.27(0.23; 0.30)	0.18	–	–	–
TBW* (kg)	23.80 ± 4.18	28.06 ± 1.42	33.28 ± 4.02	< 0.001	< 0.001***	0.004***	0.051***
TBW* (%)	60.72 ± 7.6	54.98 ± 5.03	55.41 ± 6.13	0.098	–	–	–
FFMI**				< 0.001	< 0.001****	0.002****	0.007****
(kg/m^2^)	12.5(11.1; 14.4)	15.1(13.9; 15.7)	16.3(15.7; 16.9)				
FMI* (kg/m^2^)	3.39 ± 1.52	5.46 ± 1.83	5.83 ± 1.76	0.006	0.006***	1.000***	0.047***

* = ANOVA (difference of means; normal distribution)

** = Kruskal Wallis (difference of medians; non normal distribution)

*** = Bonferroni correction

**** = Man-Whitney with Bonferroni correction; *ANact* Active anorexia, *ANrec* Recovered anorexia, *HI* Healthy individuals *BMI* Body mass index;

*R* Resistance, *Xc* Reactance, *PA* Phase angle

*FFM* Fat free mass, *FM* Fat mass

*TBW* Total body water, *FFMI* Fat free mass index, *FMI* Fat mass index significant results are highlighted by bold characters

Means ± standard deviation Medians (95% confidence interval)

Bivariate regression was performed to explore the correlation between PA and RMR.

## Results

In this preliminary study 19 women with recovering (AN_act_, *n* = 9) or active AN (AN_rec_, *n* = 10) and 18 HI were invited between May and November 2019. One patient from the AN group was excluded due to altered thyroid function and two participants from the control group were excluded due to constitutional thinness (BMI: 18.1 kg /m^2^ and 18.3 kg/m^2^). The final distribution of patients was as follows: AN_act_: *n* = 9; AN_rec_: *n* = 9; HI: *n* = 16.

Two patients with BMI value < 18.5 kg /m^2^ were classified as recovered because they had weight adequacy above 85% of the ideal weight, significant improvement in key symptoms and weight stability. Two patients with BMI > 18.5 kg/m^2^ were classified with ANact due to the intensity of key symptoms and the speed of weight loss.

Among patients with ANact amenorrhea was present in 5 (55.6%) with a median duration of 24 months; three patients (33.3%) used contraceptives and in one AN had begun in prepubertal stage (11.1%). Dietary restriction was present in all ANact patients, but 2 presented a history of diuretic use and 1 of excessive physical activity. The use of psychiatric medication (antidepressant, benzodiazepines, antipsychotics, mood stabilizer and anticonvulsant) was present in 15 patients (6 in ANact and all in the ANrec).

In univariate analysis, see Table 1, the BMI (kg/m^2^) measurement did not allow to distinguish between the ANrec x HI group. However, this distinction was observed for R, PA, FFM (kg), TBW (kg) and FFMI. The distinction between ANact x ANrec was obtained with BMI, R, PA, FFM (kg), FFMI and FMI. As expected, most tests in the univariate analysis showed the ability to discriminate the ANact x HI group.

Regarding the ordinal logistic regression with the ANact, ANrec and HI outcomes, 4 groups of independent variables with Spearman correlation values below

0.7 were analyzed, but only two of them fulfilled the assumptions of a good model fit and of non-significant test of parallel lines. The first group of variables included R, and PA and the other group included FFM (%), TBW (%) and PA. PA remained as a significant independent variable in both models (Table 2), indicating that increases in PA are related to higher probabilities of moving from ANact, to ANrec and to HI group, respectively. R remained as a significant independent variable in the first model, implying that increases in *R* are related to the probability that outcomes will move in the opposite direction (from HI to ANact). In the second group, the TBW remained at the end of the model together with the PA and the result of its increase goes in the same direction as the phase angle.

**Table 2:** Ordinal regression results

Model	Variables	OR	95% CI	*p*	Deviance	TPL
1	R(Ω)	1.015	1.003; 1.027	0.018	0.598	0.750
	PAº	0.123	0.030; 0.503	0.004		
2	PAº	0.091	0.016; 0.528	0.008	0.453	0.657
	TBW (kg)	0.524	0.345; 0.794	0.002		

*TLP* Test of parallel lines

Variables were analyzed with ROC curves in order to evaluate their capacity to distinguish between ANrec and HI individuals. PA (AUC = 0.792; CI = 0.564-1.000; *p* = 0.017) showed a good discriminatory capacity.

In relation to RMR medians, it is possible to observe a significant difference between ANact and ANrec (864.00 kcal vs 1325.00 kcal, *p* = 0.037) and ANact and HI (864.00 kcal vs 1555.50 kcal, *p* = 0.000), but no significant difference between ANrec and HI (1325.00 kcal vs 1555.50 kcal; *p* = 0,576), as evaluated by pairwise Kruskal- Wallis test adjusted by the Bonferroni correction.

All patients in ANact (778–1210 kcal; *n* = 9) are located below the 25th percentile of HI (<1361 kcal); 66.7% of ANrec (792–1339 kcal; *n* = 6) are also in this range, 11.1% (1368 kcal; n = 1) between the 25th and 75th percentile points (13601–1642 kcal) and 22.2 % (1757–1973 kcal; *n* = 2) above the 75th percentile (> 1642 kcal).

**Fig. 1:**
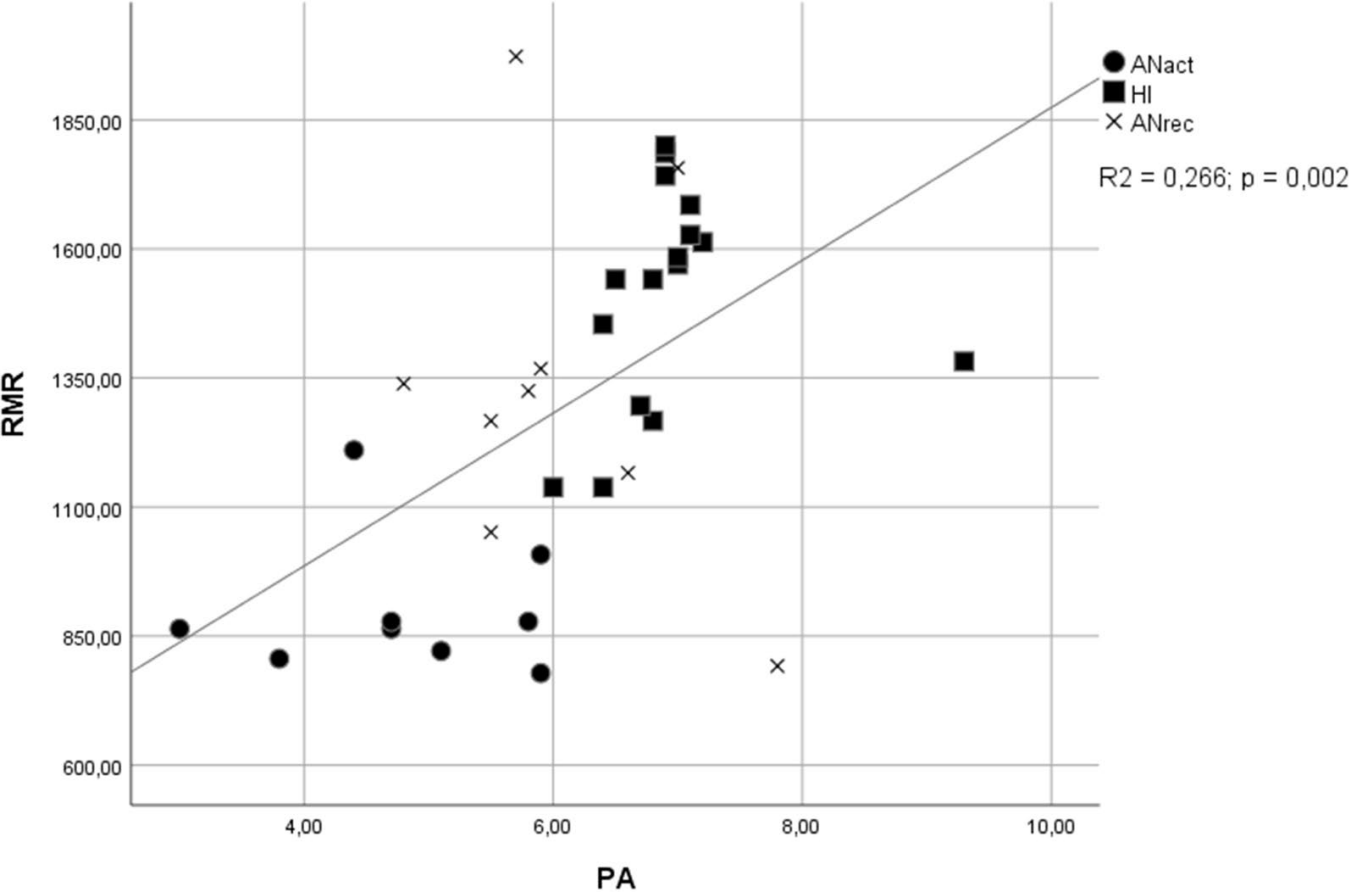
Bivariate correlation between phase angle and resting metabolic rate. ANact = patients with active anorexia; ANrec = patients with recovering anorexia; HI = health individuals;RMR = resting metabolic rate; PA = phase angle

PA and RMR displayed a significant correlation in bivariate regression (R2= 0.266; *p*= 0.002, figure 1). When observing the graph, it is visually evident that the correlation is maintained until PA values of around 7, where PA approaches its physiologic ceiling and the linearity is no longer verified. It’s also possible to observe the concentration of patients of each group (ANact, ANrec and HI) in different areas of the plot, possibly corresponding to different correlations between PA and RMR, and a greater variability among ANrec cases.

## Discussion

Recovered AN patients need a better characterization, since BMI and weight normalization may not reflect recovery of body compartments, energy expenditure and physiological condition [15, 16, 26]. The present preliminary data are part of an effort to investigate easily obtainable parameters that may be of help in this task.

The PA values in patients with AN, as demonstrated by other authors [13, 15, 26] are similar to those found in malnourished or cachexia patients [12]. In the present study, PA demonstrated a good capacity for distinguishing ANact, from ANrec and from HI, as analyzed by the regression model performed to compare the 3 study groups. There was an increase in the medians from the ANact group (4.7º; CI 4.1–5.9) to the ANrec group (5.8º; CI 5.5–6.8) and to the HI group (6.9º; IC:6.6–7.1), indicating the existence of a correlation of PA with nutritional status. The results point to a different behavior of PA in the groups, related to the persistence of nutritional/cellular changes, even in recovery. This behavior was also observed in other studies of individuals who achieved normal BMI [15, 16, 26]. Fortunato [15], showed that AN girls displayed significant differences of BMI and PA after 1 year of outpatient multidisciplinary treatment, in comparison to admission levels from, respectively, 15.18 kg/m^2^ and 4.97º to 17.42 kg/m^2^ and 5.61º. Differences of PA according to AN subtypes have also been observed, with higher levels in patients that resort to excessive physical exercise to lose weight [27]. Even though age differences for PA are largely recognized, lower PA values observed in AN_act_ and AN_rec_ cannot be assigned to the age range observed in the study. When one analyzes normal values from previous studies, it is possible to verify that PA values of younger ages aren’t as low as observed in AN_act_ and AN_rec_ groups of the present study. Additionally, PA maintained statistical significance in the both ordinal logistic regression models whose adequacy assumptions allowed analysis [12].

On the other hand, greater caution is required when it comes to BIA indirect parameters that employ anthropometric measures (e.g. weight), due to the important differences in body compartments of women along the age range of AN_act_ patients. With this remark in mind, it may be worth commenting on some of the findings related to body composition, especially in the comparison between AN_rec_ and HI subjects. BMI and FM were not different in these groups. AN_rec_ patients showed lower FFM, suggesting that FM is the main component of recovery. The FFMI, which has already been highlighted both for its clinical importance in assessing nutritional risk [28] and for its practicality of execution, suffers from the same limitation related to the age range. However, important differences between the three study groups were showed Table 1, which might be an indication of its usefulness in monitoring recovery.

The behavior of the BMI in the ANrec, is similar to that of the HI, showing its low sensitivity to changes in body composition. In the present study, the FFM showed important changes in ANrec, and the FM was characterized by displaying a similar content in relation to the patients in the HI group. Patients in ANrec seem to recover part of their FFM, which is higher than in ANact, but do not reach HI levels [2]. Iketani

[29] evaluated 20 patients with AN using dual-energy X-ray absorptiometry (DEXA) and showed, after weight recovery, a larger compartment of FM compared to the FFM. Skinfold evaluation of AN patients showed that after 13 weeks of treatment, patients gained more FM than lean mass [30].

Another finding regarding PA was the suggestion of its relation to RMR, according to bivariate regression. RMR is decreased in AN because of a reduction in metabolically active tissues and probably because of adaptation to chronic underfeeding. Since PA levels may be influenced by body cell mass, where most of the metabolic processes occurs, by derangements in cell membranes, and changes in extra- cellular fluids, the relationship of PA with metabolic activity should be explored. There seems to be, according to what has been suggested, a relationship between PA and size or the activity of metabolically active tissues [16].

Besides the limitations related to the age range in AN_act_, other important limitations have an impact on the generalization of the data presented. The one related to the sample size indicates the need for the present findings to be replicated in larger samples before any policy recommendations can be considered. However, this limitation is not easy to overcome and is related to difficulties in recruiting patients who meet the inclusion criteria or to the low compliance of AN patients to take part in studies. This problem has been described by others who show dropout rates at 25% [15].

The use of psychiatric medications by the majority of AN_rec_ patients constitutes another limitation, since they can influence the weight gain. Also, the outpatient setting could be a problem, since subjects were free to eat and take medications and, in some cases, exercise purgative practices [16, 31–33]. However, this represents the real world situation in a setting where hospital beds and admissions pose an important limitation to the care of AN patients and to the achievement of representative sample sizes, considering, in addition, the recognized difficulty for AN patients to adhere to treatments and studies.

## Conclusion

The preliminary results obtained in this study indicate that PA may be able to distinguish between patients with active AN, recovering AN and normal patients and should have its potential explored as a tool in monitoring recovery in AN. PA displayed a significant correlation with energy expenditure in bivariate analysis and should also be explored as a possible tool in the follow up of patients with AN.

Important changes in body composition were observed, both in individuals with active AN, as expected, but also in recovered patients with normalized BMI. The interpretation of these data must be viewed with care because of the age range in the group of patients with active anorexia. Nevertheless, the important differences observed may indicate that it may be worth exploring the usefulness of these parameters in monitoring recovery.

## Data Availability

The datasets used and/or analysed during the current study are available from the corresponding author on reasonable request.

## Abbreviations

AN: Anorexia nervosa
ANact: Active anorexia
ANrec: Recovered anorexia
HI: Healthy individuals
BIA: Bioelectrical impedance analysis
BMI: Body mass index
DEXA: X-Ray dual energy absorptiometry
DSM-V: Diagnostic and statistical manual of mental disorders V
ESPEN: European society for clinical nutrition and metabolism
FM: Fat mass
FMI: Fat mass index
FFM: Fat-free mass
FFMI: Fat free mass index
PA: Phase angle
ROC: Receiver operating characteristic
RMR: Resting metabolic rate
SPSS: Statistical package for social science
TBW: Total body water
TSH: Thyroid stimulating hormone
WHO: World health organization
